# Iron-Oxide-Nanoparticles-Doped Polyaniline Composite Thin Films

**DOI:** 10.3390/polym14091821

**Published:** 2022-04-29

**Authors:** Bogdan Butoi, Carmen Steluta Ciobanu, Simona Liliana Iconaru, Constantin Cătălin Negrilă, Madalina Andreea Badea, Mihaela Balas, Anca Dinischiotu, Gabriel Predoi, Bogdan Bita, Andreea Groza, Daniela Predoi

**Affiliations:** 1 National Institute for Laser, Plasma and Radiation Physics, 409 Atomistilor Street, P.O. Box MG 36, Magurele, 077125 Bucharest, Romania; bogdan.butoi@inflpr.ro (B.B.); bogdan.bita@inflpr.ro (B.B.); 2 National Institute of Materials Physics, Atomistilor Street No. 405A, P.O. Box MG 07, Magurele, 077125 Bucharest, Romania; ciobanucs@gmail.com (C.S.C.); simonaiconaru@gmail.com (S.L.I.); catalin.negrila@infim.ro (C.C.N.); 3 Department of Biochemistry and Molecular Biology, Faculty of Biology, University of Bucharest, 91–95 Splaiul Independentei, 050095 Bucharest, Romania; madalina-andreea.badea@bio.unibuc.ro (M.A.B.); mihaela.balas@bio.unibuc.ro (M.B.); anca.dinischiotu@bio.unibuc.ro (A.D.); 4 Faculty of Veterinary Medicine, University of Agronomic Sciences and Veterinary Medicine of Bucharest, 105 Splaiul Independentei, Sector 5, 050097 Bucharest, Romania; gabrielpredoi2017@gmail.com

**Keywords:** PANI, iron oxide, thin films, biocompatibility, Caco-2 cells, morphology, chemical composition

## Abstract

Iron-oxide-doped polyaniline (PANI-IO) thin films were obtained by the polymerization of aniline monomers and iron oxide solutions in direct current glow discharge plasma in the absence of a buffer gas for the first time. The PANI-IO thin films were deposited on optical polished Si wafers in order to study surface morphology and evaluate their in vitro biocompatibility. The characterization of the coatings was accomplished using scanning electron microscopy (SEM), Fourier-transform infrared spectroscopy (FTIR), atomic force microscopy (AFM), metallographic microscopy (MM), and X-ray photoelectron spectroscopy (XPS). In vitro biocompatibility assessments were also conducted on the PANI-IO thin films. It was observed that a uniform distribution of iron oxide particles inside the PANI layers was obtained. The constituent elements of the coatings were uniformly distributed. The Fe-O bonds were associated with magnetite in the XPS studies. The surface morphology of the PANI-IO thin films was assessed by atomic force microscopy (AFM). The AFM topographies revealed that PANI-IO exhibited the morphology of a uniformly distributed and continuous layer. The viability of Caco-2 cells cultured on the Si substrate and PANI-IO coating was not significantly modified compared to control cells. Moreover, after 24 h of incubation, we observed no increase in LDH activity in media in comparison to the control. In addition, our results revealed that the NO levels for the Si substrate and PANI-IO coating were similar to those found in the control sample.

## 1. Introduction

In the last years, an increased interest has been shown by the scientific community toward conductive polymers, especially polyaniline (PANI), for their physical and chemical properties such as: high conductivity, chemical stability, ion exchange capacity, and adhesion [1,2,3,4,5,6,7].

PANI can be fabricated as powders, aqueous dispersions, films, or polymer matrices for embedding nanoparticles, and it can be involved in various copolymerization synthesis procedures for improving the conductivity of final compounds [1,2,3,4,5,6,7]. For example, by doping PANI with HCl [8] or graphene and carbon nanowalls [5], the conductivity and elasticity of coatings can be enhanced. Moreover, PANI possesses antibacterial activity, and when it is used in hydrogels in combination with carboxymethyl cellulose [9], it efficiently removes microbes and dyes from waste waters. TiO_2_/PANI composites have antibacterial and antifungal properties [10]. The combination of PANI with silver nanoparticles inhibited the development of *S*. *aureus*, *S. typhi*, *E. coli*, and *P. aeruginosa* [11].

PANI is used for many applications such as: electrostatic dissipation, electrodes for sensor applications, conductive films for light emitting diodes (LEDs), anticorrosive layers for covering and protecting metals, or for conductive textiles [1]. By doping PANI with HCl or H_2_SO_4_ [8,12] or graphene and carbon nanowalls [5], its conductivity or elasticity can be increased, making it possible to develop more applications. In the paper reported by Fuseini M. et al. [6], the corrosive resistance of copper sheets was improved by electrophoretic deposition of PANI on their surfaces with an efficiency of 92.92%. Other applications of PANI include improved detection of sensors for lead [12] or electrode materials for supercapacitors [3,13].

Moreover, PANI has been investigated for its uses in biomedical applications such as biosensors, neural probes, tissue engineering, and controlled drug delivery [14,15]. Considering the potential of this conducting polymer in biomedicine, the biocompatibility of PANI has been studied in several in vitro and in vivo systems. By comparison with polypyrrole (PPy), the most studied conductive polymer, PANI showed at least a comparable potential for biomedical applications and promising biocompatibility [15,16]. Both PPy and PANI have been shown to support adhesion and cell growth and provide proliferation and differentiation in vitro. Studies were performed on a large variety of cell types from humans (neuroblastomas, cardiac myoblasts, osteosarcomas, keratocytes, Jurkat T lymphocytes, mesenchymal stem cells, endothelial cells, and bone-marrow-derived stem cells) and rodents (rat pheochromocytomas, mouse embryonic fibroblasts, and mouse hepatomas) [15,17,18,19]. However, other investigations have reported poor adhesion and growth of human immortalized nontumorigenic keratinocytes and hepatocellular carcinoma cell lines in the presence of PANI hydrochloride and a PANI base [19] and reduced biocompatibility of PPy with human mesenchymal stem cells, endothelial cells, human lung fibroblasts, and mouse alveolar macrophages [15]. In rodent models, studies stated that PPy did not activate an inflammatory or allergic response and induced only a minimal tissue response with no significant long-term effects. Moreover, PPy did not cause hemolysis or changes in mice’s blood coagulation [15,20]. On the other hand, results regarding the biocompatibility of PANI in vivo are mixed. For example, PANI-coated polycaprolactone scaffolds implanted in the backs of rats for up to 4 weeks revealed no systemic or neurological toxicity, but the presence of fibrous tissue surrounding the scaffold was noted. In addition, a minimal, mild, or moderate inflammatory response that decreased over time after implantation was reported [21]. Regarding the toxicity of this polymer toward the major target organs of acute oral toxicity, low doses of PANI nanofibers and nanospheres generated no abnormal histopathological changes in mice kidneys and liver. Nevertheless, a slight liver lesion was observed in mice exposed to a higher PANI nanofibers dose (100 mg/mL) [22]. Similar to PPy, the different biological responses to PANI are a result of its properties including conductivity, surface energy (hydrophobicity), surface roughness, mechanical actuation, dopant retention, and impurities, and they are also a result of different preparation protocols and compositions [15].

The polymerization of aniline monomers for the production of polyaniline in various chemical states, structures, or morphologies is performed by chemical, electrochemical, or plasma methods. The injection of monomers in plasma implies ionizations and electron impact dissociation processes, leading to active species formation and their coupling by a free radical polymerization mechanism [1]. Therefore, the combination of radicals in plasma leads to the generation of cross-linked polymers with high molecular weights.

In our previous paper [1], we showed that DC glow discharges can be successfully used for the generation of PANI thin films with crystalline domains embedded in amorphous matrices. The generation of PANI in emeraldine salt form conferred high conductive properties on the layers.

Over time, the incorporation of iron oxide nanoparticles in polymer matrices led to the production of nanocomposites with magnetic properties. Magnetic nanoparticles such as magnetite or maghemite have biological activity and biocompatibility and are broadly used for biomedical applications in hyperthermia cancer treatments, magnetic resonance imaging, and magnetic drug targeting of cells [23]. Several studies [23,24,25,26,27,28] reported the synthesis of polyaniline with iron oxide nanoparticles for the generation of nanocomposites with improved magnetic and conducting properties: nanocomposites of polyaniline and different iron oxides [25], graphene/polyaniline/Fe_3_O_4_ nanocomposites with superparamagnetic properties [24], and polyaniline–chitosan nanocomposites with embedded silver [26]. In the work performed by Sriramprabha R. et al. [27], Fe_2_O_3_/PANI nanocomposite electrodes improved the sensitivity and selectivity of sensors for creatinine detection. They showed that by using such electrodes, there was no more need for any bioreceptors or binders in real-time CRE quantification. Fe_2_O_3_/PANI composites have also been studied for their anticorrosive properties when covering steel substrates [4]. Prepared by oxidative polymerization, in a 1:1 ratio, Fe_2_O_3_/PANI composites have higher corrosive resistance than plain materials [4].

This work concentrates on the first development of iron-oxide-doped polyaniline (PANI-IO) composite thin films obtained in direct current glow discharge plasma in the absence of a buffer gas. Structural and morphological analyses were conducted using scanning electron microscopy (SEM), Fourier-transform infrared spectroscopy (FTIR), atomic force microscopy (AFM), metallographic microscopy (MM), and X-ray photoelectron spectroscopy (XPS). The biological activity of the PANI-IO composite thin films was also reported.

## 2. Materials and Methods

### 2.1. Materials

Aniline precursors in liquid form (Sigma-Aldrich Chemistry, Dorset, UK) were used as precursors for generation of thin polymeric films by plasma polymerization in a DC glow discharge reactor. For the synthesis of iron oxide, we used precursors including ferrous chloride tetrahydrate (FeCl_2_·4H_2_O), natrium hydroxide (NaOH), ferric chloride hexahidrate (FeCl_3_·6H_2_O), hydrochloric acid (HCl), and perchloric acid (HClO_4_) that were acquired from Merck (Darmstadt, Germany) and deionized water.

### 2.2. Synthesis of Iron Oxide Nanoparticles

The iron oxide was obtained using the most standard method, coprecipitation. The ferric and ferrous ions were mixed in a 1:2 molar ratio at room temperature in a basic solution in accordance with previous studies [29].

### 2.3. Deposition Method for Iron-Oxide-Doped Polyaniline

**Figure 1:**
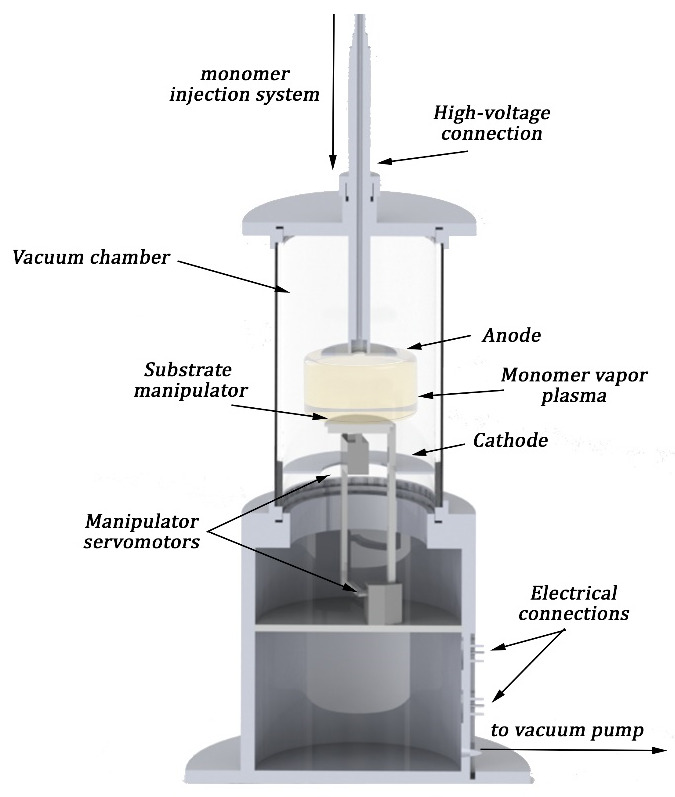
Scheme of the deposition setup.

In order to obtain thin iron-oxide-doped polyaniline films, a setup made up of a vacuum pump, vacuum chamber (3000 cm^3^), two parallel mounted inner circular electrodes, and an inlet system were used. A similar setup was involved in the generation of PANI films with different morphological and structural proprieties [1]. A schematic representation of the deposition setup is shown in Figure 1.

Inside the deposition chamber, centrally between the two electrodes, a sample holder containing 8 Si (100) wafer substrates (15 × 12 mm^2^) was introduced. The setup allowed either the grounding or voltage biasing of the substrate holder, but the PANI-IO films were produced at floating potential in order to not disturb the electrical field of the plasma that contained iron oxide nanoparticles. After reaching a base pressure of around 5 × 10^−3^ mbar, a mixture of aniline liquid precursors with iron oxide nanoparticle solution were injected inside the deposition chamber through a hole in the anode. By applying 1500 V and a discharge current of 30 m between electrodes, the DC glow discharge was ignited and the polymerization process started. The working pressure was kept at around 5 × 10^−1^ mbar by controlling the injection flow. The deposition time was 10 min with a deposition rate of 1.5 nm/s, which corresponded to a total film thickness of 1 micron.

### 2.4. Characterization Methods

The topology of the polyaniline coatings deposited onto Si substrates was investigated by scanning electron microscopy (SEM) using a ThermoFisher Apreo S scanning electron microscope (ThermoFisher, Hilsboro, CA, USA) in both high- and low-vacuum modes.

The IR spectra of the polyaniline layers obtained on the Si substrate were acquired in the spectral range of 4000–400 cm^−1^ using a SP100 IR Perkin Elmer spectrometer (Waltham, MA, USA) equipped with an attenuated total reflection (ATR) accessory.

X-ray photoelectron spectroscopy (XPS) studies were performed using a SPECS XPS spectrometer, PHOIBOS 150 analyzer, monochrome RX source (300 W), and Al Kα (1486.61 eV). The acquisition was made with an energy pass of 20 eV for spectral lines and 50 eV for the extended spectrum.

Metallographic microscopy (MM) studies were conducted with the aid of an inverted trinocular metallographic microscope (OX.2153-PLM, (Euromex, Arnhem, The Netherlands)). The microscope was equipped with a CMEX digital camera (1.3 MP), and the MM images (20× objective) were acquired in ambient conditions using ImageFocusAlpha software (v 1.3.7.19728, Euromex, Arnhem, The Netherlands). In addition, ImageJ software [30] was used for the obtaining of 3D representations of MM images.

The surface topography of the PANI-IO thin films was investigated using atomic force microscopy (AFM) technique with an NT-MDT NTEGRA Probe Nano Laboratory instrument (NT-MDT, Moscow, Russia). The studies were performed at room temperature in noncontact mode employing a silicon NT-MDT NSG01 cantilever (NT-MDT, Moscow, Russia) coated with a 35 nm gold layer. The AFM data were recorded on a surface of 30 × 30 µm^2^ and were processed with the aid of the dedicated software Gwyddion 2.59 (Department of Nanometrology, Czech Metrology Institute, Brno, Czech Republic) [31]. The roughness parameter known as root mean square roughness (R_RMS_) was also determined from the AFM data.

### 2.5. In Vitro Biocompatibility Assessment

#### 2.5.1. Caco-2 Cell Line

Caco-2 cell line (HTB-37™), which is represented by human epithelial cells isolated from colon tissue, was purchased from ATCC (American Type Culture Collection, Manassas, VA, USA). The cells were grown in 75 cm^2^ culture flasks and Minimum Essential Medium (MEM, 61100-087, Gibco, Invitrogen, Paisley, Scotland, UK) supplemented with 1.5 g/L NaHCO_3_, 1 mM sodium pyruvate (11360-039, Gibco, Carlsbad, CA, USA), 1% penicillin/streptomycin/amphotericin B mix antibiotic–antimycotic solution (A5955, Sigma-Aldrich, St. Louis, MO, USA), and 20% fetal bovine serum (10270-106, origin of South America, Gibco, Life Technologies, Carlsbad, CA, USA). Caco-2 cultures were preserved at 37 °C in a humidified atmosphere (95%) with 5% CO_2_. The culture medium was completely refreshed every two days, and for subcultivation, a 0.25% trypsin-0.53 mM EDTA solution was used.

#### 2.5.2. Caco-2 Cell Culture on Si Substrate and PANI-IO Coating

For the evaluation of biocompatibility, Si substrate and PANI-IO coating were placed in 24-well plates. Caco-2 cells were seeded on top of them at a density of 7 × 10^4^ cells/mL and incubated for 24 h. Cells cultured on the surface of a 24-well plate were used as controls. Before culturing, Si substrate and PANI-IO coating were sterilized by UVC radiation for 1 h.

#### 2.5.3. MTT Cell Viability Test

After incubation of cells with Si substrate and PANI-IO coating for 24 h, cell viability was evaluated by MTT test. For this purpose, the culture medium was discarded, and Caco-2 cells were incubated with 500 μL of a 1 mg/mL MTT (3-(4,5-dimethylthiazol-2-yl)-2,5-diphenyltetrazolium bromide) solution (M5655, Sigma-Aldrich, St. Louis, MO, USA) for 2 h at 37 °C. Afterward, the MTT solution was removed, and the formazan crystals that formed as a result of mitochondrial dehydrogenase activity were solubilized with 250 μL isopropanol. Finally, the optical density was read with a Tecan GENios microplate reader (Tecan Trading AG, Männedorf, Switzerland) using a 595 nm wavelength. The results were expressed in percentages related to control (100% viability).

#### 2.5.4. Lactate Dehydrogenase (LDH) Assay

The level of lactate dehydrogenase (LDH) released in culture medium was estimated after 24 h using a commercial kit from Roche (Cytotoxicity Detection Kit (LDH), cat. No. 11644793001, Basel, Switzerland) as an indicator of cell membrane integrity. This method is based on the reduction of NAD^+^ to NADH/H^+^ by the LDH-catalyzed conversion of lactate to pyruvate and the formation of formazan salt as a result of H/H^+^ transfer from NADH/H^+^ to tetrazolium salt INT (2-[4-iodophe-nyl]-3-[4-nitrophenyl]-5-phenyltetrazolium chloride). Briefly, 50 μL of culture medium was homogenized with 50 μL of catalyst (diaphorase/NAD^+^ mixture) and dye (INT and sodium lactate) mix solution in a 96-well plate. After 20 min of incubation at room temperature, in dark conditions, the absorbance was measured at 485 nm.

#### 2.5.5. Nitric Oxide (NO) Production Measurement

The release of nitric oxide (NO) from Caco-2 cells after 24 h exposure to Si substrate and PANI-IO coating was evaluated using the Griess method as an indicator of an inflammatory response. Thus, 80 μL of culture medium was mixed with 80 μL Griess reagent (0.1% N-(1-naphthyl)ethylenediamine: 1% sulfanilamide in 85% phosphoric acid 1:1), and the optical density of the azo dye formed in Griess reaction was read at 550 nm in a 96-well plate. The amount of NO released in the culture medium was calculated using a 0–100 μM NaNO_2_ standard curve.

#### 2.5.6. F-Actin Cytoskeleton Labeling

Actin filaments (F-actin) were studied to evaluate the cytoskeleton integrity of Caco-2 cells cultured on Si substrate and PANI-IO coating for 24 h. After this period, the culture medium was discarded, and the cells were fixed with a 4% paraformaldehyde solution for 20 min at 4 °C. Then, the cells were washed with phosphate buffer saline (PBS) and permeabilized with a 0.1% TRITON X-100 + 2% BSA solution in PBS for 45 min at room temperature. Labeling of F-actin filaments was obtained by incubation of cells with a 150 nM Alexa Fluor™ 488 phalloidin (A12379, Life Technologies Co., Eugene, OR, USA) solution (prepared in PBS solution containing 1.2% bovine serum albumin) for 45 min at room temperature in dark conditions. Furthermore, the cells were washed with PBS and then incubated with 2 μg/mL Hoechst 33342 (H3570, Molecular probes by Life Technologies, Eugene, OR, USA) solution (10 min, room temperature, dark conditions) for nuclei staining. In the end, the cells were visualized with an Olympus IX 71 fluorescence microscope (Olympus, Tokyo, Japan) on green and blue filters, and the images were captured using the Cell F software (Version 5.0, Olympus, Tokyo, Japan).

#### 2.5.7. CMFDA Staining for Intracellular Glutathione (GSH)

Intracellular glutathione (GSH) was stained with CellTracker™ Green 5-chloromethylfluorescein diacetate fluorescent dye (CMFDA, C2925, Life Technologies Co., Eugene, OR, USA). Thus, the culture medium was discarded, and the cells were incubated with a prewarmed 10 μM CMFDA solution prepared in serum-free medium (30 min, 37 °C). After this step, the CMFDA solution was removed. Finally, the cells were fixed with a 4% paraformaldehyde solution (15 min, room temperature), washed with PBS, and then visualized with an Olympus IX 71 fluorescence microscope (Olympus, Tokyo, Japan). The images were acquired using FITC green filter and Cell F software (Version 5.0, Olympus, Tokyo, Japan). CMFDA fluorescence of total GSH content and nuclear GSH was quantified using ImageJ software (v1.52a, National Institutes of Health, Bethesda, MD, USA) [32] and calculated as corrected total cell fluorescence (CTCF) based on the following formula: CTCF = Integrated Density − (Area of selected cell × Mean fluorescence of background readings). Cytoplasmic GSH was obtained via the difference between total and nuclear GSH and expressed as percentages related to control. A total of 20 cells from 5 different images were analyzed for each experimental condition.

#### 2.5.8. Statistical Analysis

The experiments were performed in triplicate, and data were calculated as mean values ± standard deviation (SD) and expressed in percentages related to control. The statistical significance between the experimental groups and control was determined by the Student’s *t*-test. The results were significant for * *p* < 0.05, ** *p* < 0.01, and *** *p* < 0.001.

## 3. Results and Discussion

**Figure 2:**
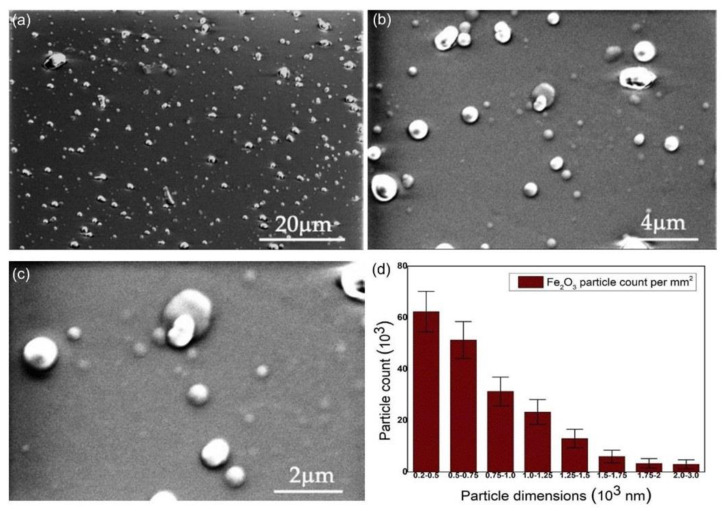
SEM images of PANI-IO film obtained by DC plasma polymerization at: (**a**) 2500×, (**b**) 10,000×, (**c**) and 20,000× magnifications. (**d**) The particle size distribution of iron oxide particles in PANI layers.

**Figure 3:**
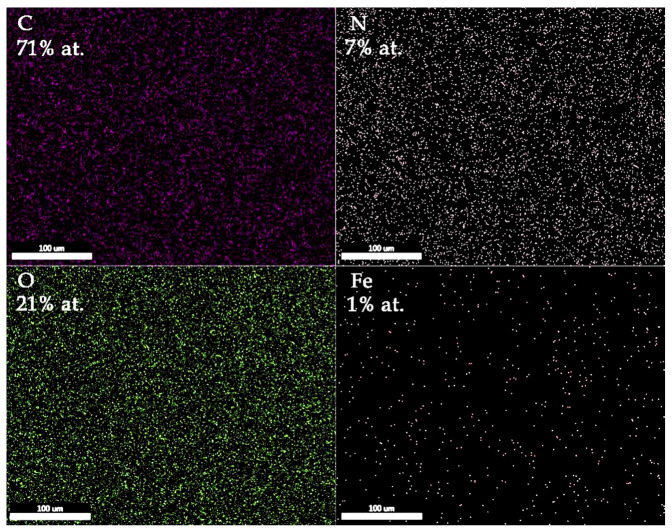
SEM-EDS elemental mapping of PANI-IO thin film.

With SEM analysis, information about the morphology of the maghemite-doped polyaniline (PANI-IO) thin films deposited on optical polished Si wafers was revealed. The images of PANI films acquired at 2500×, 10,000×, and 20,000× magnifications are presented in Figure 2. The SEM image from Figure 2a presents the uniform distribution of iron oxide particles inside the PANI layers. At a close look (see Figure 2c), the dimensions of the iron oxide particles embedded into the polymeric film ranged from 200 nm to 3 µm. In Figure 3, it can be observed that a higher number of particles ranged between 200 and 750 nm in diameter.

The SEM-EDS elemental mapping results of the PANI-IO coating are presented in Figure 3. The C, N, O, and Fe elements were uniformly distributed on the analyzed area of 100 × 100 µm^2^. The atomic concentrations of film constituents are also highlighted in the image.

**Figure 4:**
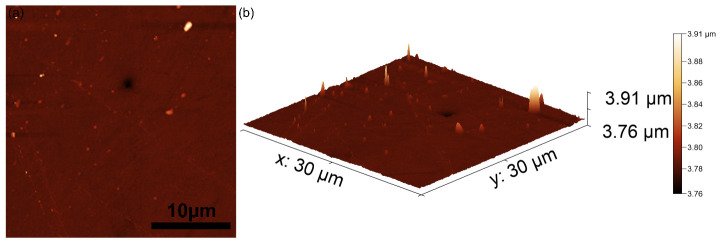
A 2D atomic force microscopy (AFM) topography image of PANI-IO thin films’ surface (**a**) and 3D representation of the surface topography of PANI-IO thin films’ surface (**b**).

Complementary complex information regarding the surface topography of the PANI-IO thin films was revealed with the aid of AFM studies. The specific 2D AFM topography of an area of 30 × 30 µm^2^ of the PANI-IO thin films is presented in Figure 4a.

The AFM 2D topography of the PANI-IO thin films’ surface depicted in Figure 4 as well as the 3D representation emphasized that the thin films exhibited the morphology of a uniform and continuous deposited layer without presenting any traces of fissures or cracks. Moreover, the 3D representation suggested that the iron oxide particles were uniformly distributed inside the PANI layers. In addition, the AFM data allowed the determination of the roughness parameter, root mean square roughness (R_RMS_), value for the PANI-IO thin films, which was 3.72 nm. The data resulting from the AFM investigation was in good agreement with the results obtained by SEM visualization.

**Figure 5:**
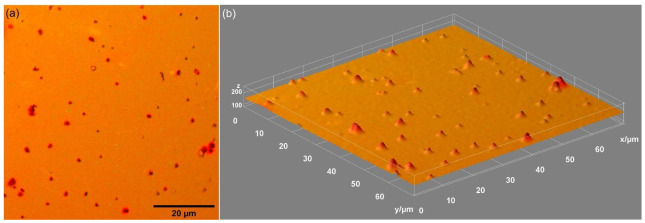
The 2D (**a**) and 3D (**b**) MM images obtained for PANI-IO thin films (20× objective).

Complementary information regarding the surface morphology of the PANI-IO thin films was provided by MM studies. The results of our MM studies are presented in Figure 5. Thus, on the Si surface, we noticed the presence of a continuous PANI-IO thin film. In both of our MM images (2D and 3D representations), surface defects (such as cracks) or other impurities were not observed. The results of the MM studies were in good agreement with those obtained with SEM and AFM.

**Figure 6:**
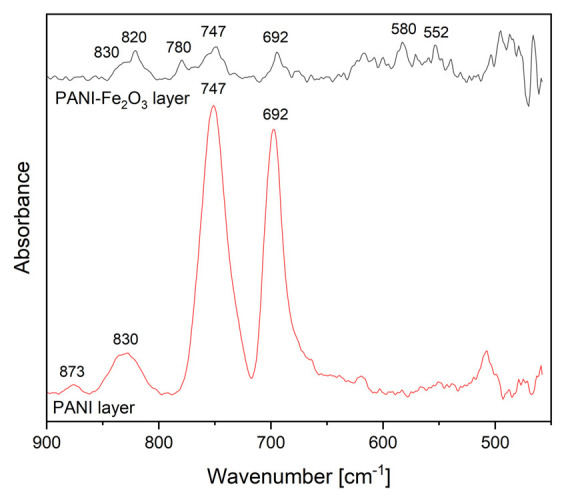
FTIR spectra of PANI (the red line) and PANI-IO (the black line) layers.

The FTIR spectra of PANI-IO and PANI layers deposited on silicon substrates are presented in Figure 6. Usually, the metal–O–metal bonds manifest vibrations in the 650–550 cm^−1^ spectral range [28,33]. Therefore, we attributed the IR bands observed in the FTIR spectrum of the PANI-IO layer at 580 and 552 cm^−1^ to Fe-O-Fe bonds [34], while the IR band at 466 cm^−1^ was assigned to Fe-O (metal-O [28,33,34]).

In the spectral range of 1000–450 cm^−1^, we saw the major vibration modes that are characteristic to the polymerization process of PANI, namely, the ortho, meta, and para substitutions in benzene rings [1,35]. The ratio between the intensities of 747 (ortho substitutions, 1,2 disubstitutions in benzene ring) and 692 cm^−1^ (meta substitutions, 1,3 disubstitutions in benzene ring) was similar in both FTIR spectra from Figure 6. The 873 cm^−1^ IR band was due to meta substitutions in benzene rings [1,35].

The para substitutions in benzene rings manifest vibrations in the 860–800 cm^−1^ spectral range and represent a measure of the conductivity of PANI layers [1]. The band from 830 cm^−1^ assigned to para substitutions (in the FTIR spectrum of the PANI layer) appeared in the FTIR spectrum of the PANI-IO layer as a shoulder on the 820 cm^−1^ IR band, which can also be attributed to para substitutions [35]. Therefore, the diminishing of the intensity of the IR band specific to para substitutions at 830 cm^−1^ and the appearance of the IR band at 820 cm^−1^ denoted that the presence of iron oxide in the polymer structure affected its conductivity. The IR band at 780 cm^−1^ that appeared only in the FTIR spectrum of the PANI-IO layer was assigned to C-H out-of-plane bending vibrations of a phenyl ring [1,35]. The presence of the IR bands between 820 and 780 cm^−1^ in the spectrum of the PANI-IO layer signified that the simultaneous deposition of aniline and the Fe_2_O_3_ solution in plasma influenced the coupling between the phenyl nuclei and amino groups during the polymerization process of polyaniline.

**Figure 7:**
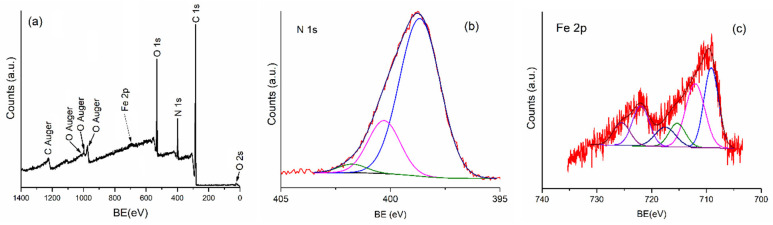
The general spectrum of PANI-IO (**a**), the N 1s XPS core-level spectrum of PANI-IO (**b**), and the Fe 2p high-resolution core-level XPS spectra of PANI-IO (**c**).

XPS studies were used for the characterization of iron oxide (PANI-IO)-doped polyaniline coatings. XPS spectra were registered to determine the phase and composition of the PANI-IO nanoparticles. The XPS general spectrum of PANI-IO (Figure 7a) revealed the presence of O 1s, N 1s, C 1s, and Fe 2p. The elements such as carbon and nitrogen belonged to polyaniline, while the element iron belonged to the iron oxide used as a dopant. Figure 7b,c show the deconvoluted N 1s and Fe 2p XPS spectra of PANI-IO thin films. The N 1s XPS core-level spectrum of PANI-IO (Figure 7b) was deconvoluted into three peaks in agreement with previous research [36]. The three peaks were centered at 398.6, 400.2, and 402.0 eV. The peak located at 398.6 eV was attributed to imine nitrogen (=N-), which was in good accordance with preceding studies [37]. According to former studies [38], the presence of an imine peak observed in the deconvoluted N 1s spectrum indicates incomplete protonation. The presence of a peak located at around 400.2 eV revealed the presence of a radical cation (-N^•+^ H-) [39]. The peak located at around 402.0 eV was attributed to generated iminium ions (-N^+^ H-).

The Fe 2p high-resolution core-level XPS spectra of PANI-IO in Figure 7 revealed the presence of a peak at 710.9 (Fe 2p3/2) and 721.98 eV (Fe 2p1/2), which were attributed to Fe^2+^. The peaks at 711.06 (Fe 2p3/2) and 724.89 eV (Fe 2p1/2) were assigned to Fe^3+^. These results were in agreement with former studies [40,41,42]. The peaks at 724.89 and 710.9 eV that were attributed to Fe 2p1/2 and Fe 2p3/2, respectively, corresponded to an Fe-O bond and were consistent with data reported for iron oxide maghemite (γ-Fe_2_O_3_) in previous studies [43].

**Figure 8:**
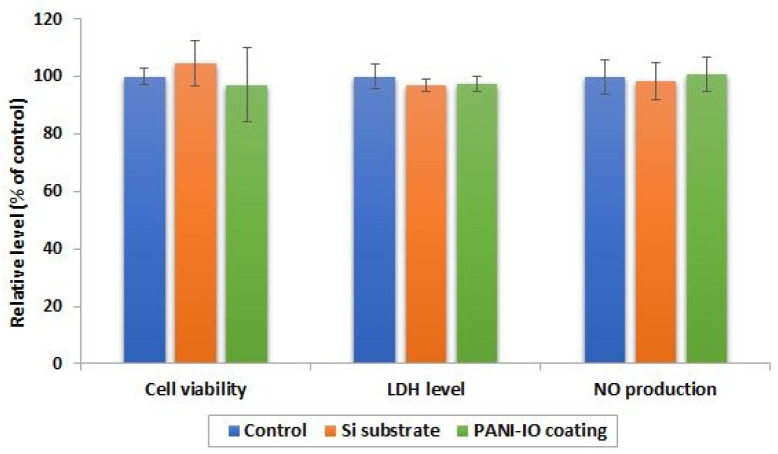
Biocompatibility of Si substrate and PANI-IO coating. The cell viability of Caco-2 cells was assessed by MTT test after 24 h from cell seeding on Si substrate and PANI-IO coating. The LDH level in the culture medium of Caco-2 cells, as a marker of membrane integrity, was estimated through an enzymatic assay based on the LDH-catalyzed conversion of lactate to pyruvate. Nitric oxide (NO) production was measured by Griess reaction method. Results were expressed as mean ± standard deviation (SD) and represented related to control (100%, untreated cells).

The biocompatibility of the Si substrate and PANI-IO coating was assessed with human cells after 24 h of incubation by evaluating the cell viability, membrane integrity, and inflammatory response induced in Caco-2 cell cultures (Figure 8).

The results showed that the viability of Caco-2 cells cultured on the Si substrate and PANI-IO coating was not significantly modified compared to control cells, registering a cell viability percentage of 104.6% and 97.1%, respectively. The values obtained indicated that no cell death or inhibition of the proliferative capacity of Caco-2 cells was triggered in the presence of the Si substrate or PANI-IO coating. Furthermore, we evaluated the membrane integrity of Caco-2 cells by measuring the activity of LDH released in the culture medium. In normal conditions, LDH is intracellularly located, but when the membrane is damaged, this enzyme is released outside the cells in the biological media. After 24 h of incubation, we observed no increase in LDH activity in media in comparison to the control. This finding confirmed the biocompatibility of the tested materials and confirmed the MTT assay results. To analyze the inflammatory response of cells to the Si substrate and PANI-IO coating, the level of NO released in the culture medium was registered. NO acts as a proinflammatory mediator that induces inflammation due to overproduction in abnormal conditions. Our results revealed no change in NO production in media of cells cultured on the Si substrate and PANI-IO coating. Considering this, we concluded that the tested materials possess good biocompatibility and are unlikely to activate inflammatory processes in human cells.

**Figure 9:**
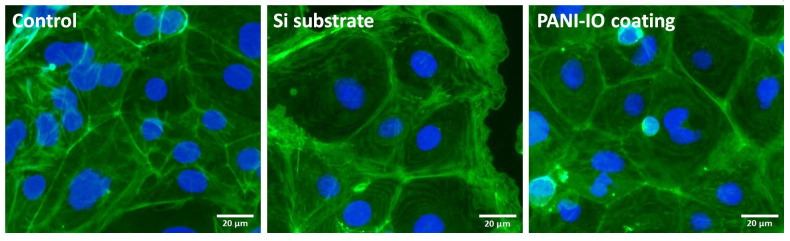
Fluorescence labeling of F-actin cytoskeleton in Caco-2 cells cultured on Si substrate and PANI-IO coating for 24 h. Green fluorescence indicates F-actin filaments staining by Alexa Fluor™ 488 phalloidin, and blue fluorescence is for nuclei (Hoechst). Control represents cells cultured in the absence of substrates. Magnification: 10×. Scale bar: 20 µm.

Cell morphology was analyzed through the fluorescence labeling of F-actin cytoskeleton. The fluorescence microscopy images presented in Figure 9 revealed that Caco-2 cells cultured on the Si substrate and PANI-IO coating preserved their epithelial-like specific morphology and maintained their capacity for adherence and spreading. Moreover, the cytoskeleton integrity and organization were not altered, and cell–cell interactions were maintained in both experimental conditions, similar to the control. Accordingly, these results indicated a high tolerance of cells toward these materials, and they did not seem to disturb the proliferative capacity and specific morphology of Caco-2 cells. Our findings are also supported by other studies. For example, Liu et al. reported that PANI films deposited over a Si substrate promote a greater cellular adhesion and proliferation of PC-12 cells than films on a Si substrate without a polymer, possibly due to the rough surface of PANI [44].

**Figure 10:**
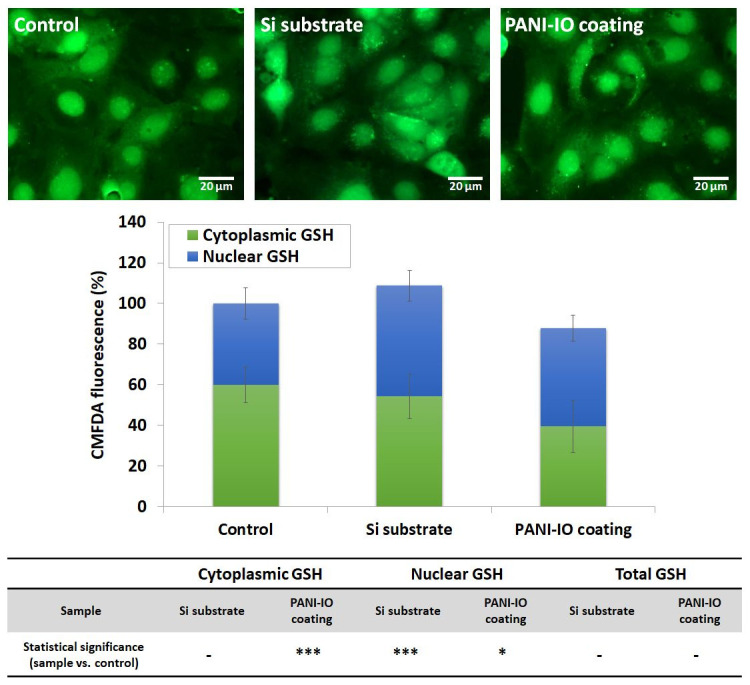
CMFDA relative fluorescence in cytoplasm and nucleus of Caco-2 cells cultured on Si substrate and PANI-IO coating for 24 h. Green fluorescence indicates GSH staining by CMFDA fluorescent dye. Control represents cells cultured in the absence of substrates. The graph is the corresponding fluorescence quantification of microscopy images presented above. The results were calculated as a percentage of the total GSH of each sample, normalized to control, and expressed as a percentage of nuclear and cytoplasmic GSH content. Magnification: 10×. Scale bar: 20 µm. Statistical significance was calculated, related to control, for each experimental condition (* *p* < 0.05 and *** *p* < 0.001 vs. control).

Cellular reduced glutathione (GSH) is the most abundant thiol antioxidant and protects cells against oxidative damage. In mammalian cells, GSH is biosynthesized in the cytosol, but it is also distributed to different intracellular organelles including the nucleus. The transport of GSH from the cytosol plays an important role in the regulation of glutathione redox homeostasis in the cytosol and other subcellular compartments [45]. In this study, the GSH content was estimated in cells incubated for 24 h in the presence of a Si substrate and PANI-IO coating by quantifying the CMFDA fluorescence of the cytosol and nucleus. As shown in Figure 10, the cytoplasmic GSH level of cells cultured on the PANI-IO coating significantly decreased (*p* = 0.0002 ***) by 20.3% compared to the control, while the nuclear GSH content increased by 8.2% (*p* = 0.01 *). A significant elevation in nuclear GSH content by 14.2% (*p* = 0.0002 ***) compared to the control was also observed in cells cultured on the Si substrate. However, we registered no significant modifications in the total GSH content of samples, related to the control, suggesting only a possible redistribution of the GSH pool between nuclear and cytoplasmic cellular compartments, without compromising cell redox status. Redistribution of GSH toward the nucleus in the presence of a PANI-IO coating might be a defense cell response to a possible perturbation of subcellular GSH redox homeostasis triggered by iron ions released from the PANI-IO coating and subjected to Fenton reactions upon cell internalization. Previously, it was reported that GSH promotes Fenton degradation by accelerating the Fe(II)/Fe(III) cycle, thus producing a higher quantity of hydroxyl radicals [46]. A recent study found that the Cu(II) ions released from iron–copper co-doped PANI nanoparticles (Fe-Cu@PANI) can also undergo redox reactions in the presence of GSH [47]. Moreover, intranuclear accumulation of GSH promotes proliferation [48]. All these might indicate that a PANI-IO coating could promote the induction of cell proliferation, thereby contributing to a beneficial tissue response and clinically relevant performance.

In summary, in vitro biological assessment demonstrated the biocompatibility of the PANI-IO coating. No decrease in cell viability or membrane or morphology alterations were induced in Caco-2 cells for up to 24 h. Moreover, the PANI-IO coating did not trigger a significant inflammatory response or oxidative damage. However, further studies are needed to gain insights into the possible links and implications in the perturbations of cellular redox status.

Lately, functionalized iron oxide nanoparticles have attracted attention due to the possibility of being used in various medical treatments and in the diagnosis of certain diseases. Our studies on obtaining thin layers of functionalized iron oxide nanoparticles are part of the considerable efforts made by researchers in recent years to be able to control the manufacturing of nanostructured materials that have specific functional properties. The use of polyaniline for the functionalization of iron oxide nanoparticles offers a great advantage. Thus PANI-IO can preserve the properties of iron oxides as well as the other properties of organic molecules [49]. The FTIR spectra of PANI-IO nanocomposites layers behaved similarly to those observed in PANI layers, showing that the iron oxide (IO) nanoparticles’ surface was coated with PANI, thereby forming a typical core–shell conformation [50]. The nanostructure of the PANI-IO nanocomposites layers was confirmed by various microscopy methods. The surface of the PANI-IO nanocomposites layers were found to have significant biocompatibility. The studies regarding the in vitro biological assessment of PANI-IO nanocomposites layers showed that they did not induce any changes in the membrane or morphology of Caco-2 cells for up to 24 h. Moreover, no decrease in cell viability was observed within 24 h. The results we obtained in this study on the surface and biocompatibility of PANI-IO layers will lead to new studies on their electrical and magnetic properties. On top of that, further studies will be conducted to obtain information on possible links and implications for cellular redox disorders. These coatings could be used to stimulate cells and tissues.

## 4. Conclusions

In summary, in this study, we reported, for the first time, iron-oxide-doped polyaniline thin films obtained by the polymerization of aniline monomers and iron oxide solutions in direct current glow discharge plasma in the absence of a buffer gas. This was a convenient method to produce PANI-IO thin films. The PANI-IO thin films fabricated by this method were homogenous with a uniform distribution of iron oxide particles inside the PANI layers. The EDS analysis confirmed the uniform distributions of all constituent elements in the surface of the coatings. The AFM studies suggested that the surface morphology of the PANI-IO thin films was homogenous, uniform, and continuous. Moreover, the 3D representation of the AFM topography highlighted that the iron oxide particles were uniformly and evenly distributed in the PANI layers. On the other hand, the XPS studies revealed that the Fe-O bonds were associated with magnetite. The in vitro biocompatibility assessment showed that the tested materials possess good biocompatibility and are unlikely to activate inflammatory processes in human cells. Moreover, the results regarding the in vitro biocompatibility assessment indicated a high tolerance of cells for PANI-IO films as they did not seem to disturb the proliferative capacity or specific morphology of Caco-2 cells.

## Data Availability

Not applicable.
